# Analysis of Expression and Its Clinical Significance of the Secreted Phosphoprotein 1 in Lung Adenocarcinoma

**DOI:** 10.3389/fgene.2020.00547

**Published:** 2020-06-12

**Authors:** Zixin Guo, Jingyu Huang, Yujin Wang, Xiao-Ping Liu, Wei Li, Jie Yao, Sheng Li, Weidong Hu

**Affiliations:** ^1^Department of Thoracic Surgery, Zhongnan Hospital of Wuhan University, Wuhan, China; ^2^Department of Biological Repositories, Zhongnan Hospital of Wuhan University, Wuhan, China; ^3^Hubei Key Laboratory of Tumor Biological Behaviors, Hubei Cancer Clinical Study Center, Zhongnan Hospital of Wuhan University, Wuhan, China; ^4^Department of Urology, Zhongnan Hospital of Wuhan University, Wuhan, China; ^5^Department of Oncology, The First People’s Hospital of Tianmen, Tianmen, China; ^6^Human Genetics Resource Preservation Center of Hubei Province, Wuhan, China

**Keywords:** secreted phosphoprotein 1, lung adenocarcinoma, prognosis, prognostic marker, osteopontin, bioinformatics

## Abstract

**Objective:**

To explore the expression of secreted phosphoprotein 1 (SPP1) in lung adenocarcinoma (LUAD), and evaluate its relationship with clinicopathological characteristics and prognosis of LUAD, and analyze the advantages of SPP1 as a potential prognostic marker in LUAD.

**Methods:**

The expression of SPP1 in normal lung tissue and LUAD was analyzed from the Cancer Cell Line Encyclopedia (CCLE), Gene Expression Omnibus (GEO), and Human Protein Atlas (HPA) databases. GSE68465 was used to explore the relationship between the SPP1 expression and clinicopathological characteristics and the prognosis of LUAD patients. The relationship between SPP1 and immune infiltration in LUAD was analyzed by the Tumor Immune Estimation Resource (TIMER) database. Gene enrichment analysis was performed in GSEA. The Cancer Genome Atlas (TCGA)-LUAD data was used to verify the results.

**Results:**

In the cell line level, non-small cell lung cancer ranked ninth among cancer cell lines based on SPP1 expression. In the messenger RNA (mRNA) and protein levels, SPP1 expression was higher in LUAD tissues than that in normal control. SPP1 expression was related to gender, N stage, histological grade, and progression or relapse. In men, SPP1 expression were higher compared to that in women. The higher the N stage, the higher the SPP1 expression level. As LUAD progresses or relapses, SPP1 expression could increase. In the pathological grade, the SPP1 expression was higher in LUAD samples with moderate differentiation. In addition, the overall 5-year survival rates of the SPP1 high and low expression groups were 50.574 and 59.181% [*P* = 0.008; hazard ratio (HR) = 0.7057; 95% CI, 0.5467–0.9109], indicating that SPP1 had an impact on overall survival for LUAD patients. The relationship between SPP1 expression and CD4^+^ T cell, macrophage, neutrophil, and dendritic cell infiltration was weak in LUAD. SPP1 could be considered as an independent prognostic marker in LUAD (*P* = 0.003; HR = 1.150; 95% CI, 1.048–1.261) by multivariate Cox regression analysis. The results of GSEA indicated that samples with high SPP1 expression were enriched in protein secretion, mTORC1 signaling, angiogenesis, and glycolysis pathway. The analysis results obtained by TCGA-LUAD data were basically consistent with the results obtained by GSE68465.

**Conclusions:**

SPP1 can not only affect the occurrence and development of LUAD but also may be an independent prognostic marker of LUAD. SPP1 is expected to be a new target for molecular targeted therapy.

## Introduction

Lung cancer is one of the malignant tumors that seriously endanger human health worldwide. The morbidity of lung cancer accounts for 11.6%, ranking first of all cancers, and 18.4% of the total number of cancer deaths are lung cancer (Bray et al., 2018). Non-small cell lung cancer (NSCLC) accounts for 80% of total lung cancer and is the most common histological type of lung cancer (Johnson et al., 2012). Lung adenocarcinoma (LUAD) accounts for ∼40% of all lung cancers, which is the most aggressive pathological type of NSCLC, and is an important cause of death from respiratory cancer (Noguchi et al., 1995). Compared with different types of lung cancers, LUAD has slower growth and no specific clinical manifestations in its early stage. It is easy to misdiagnosis because of the similar symptoms of common respiratory diseases (Lam et al., 2013; Berardi et al., 2016). Approximately 75% of patients are diagnosed with metastatic or advanced cancer and often lose the opportunity for radical surgery. With the improvements of modern clinical diagnostic methods, the survival rate is still unsatisfactory, only ∼15%, although the overall survival rate and the quality of life have been improved (Siegel et al., 2015; Franceschini et al., 2017). In recent years, in addition to improving the treatment of lung cancer patients, searching for effective tumor markers to guide clinical diagnosis has become a hot spot in LUAD research.

Secreted phosphoprotein 1 (SPP1), also known as osteopontin, is a secretory phosphorylated glycoprotein rich in arginine–glycine–aspartic acid sequence (RGD domain) (Wei et al., 2017). It is a widely expressed mucoprotein, which can be detected in normal tissues, body fluids, and cells (Liaw et al., 1998). SPP1 also participates in the regulation of physiological processes such as development, differentiation, inflammation, and the wound healing (Fedarko et al., 2001). In addition, more and more evidence showed that SPP1 was highly expressed in various kinds of tumors and plays a considerable role in the occurrence and metastasis of various tumors (Kijewska et al., 2017; Li Y. et al., 2017; Zeng et al., 2018; Zhang et al., 2018). The aim of this research was to explore the relevance between SPP1 and clinicopathological features of patients with LUAD and to determine whether it can be used as an independent prognostic marker of LUAD.

## Materials and Methods

### Cancer Cell Line Encyclopedia Analysis

Cancer Cell Line Encyclopedia (CCLE^1^) is a database of cancer cell lines maintained by the Broad Institute of MIT & Harvard, which is an open-access database including the RNA-Seq data for the current 1,457 cancer cell lines (Barretina et al., 2012). We used CCLE database to analyze the messenger RNA (mRNA) expression levels of the SPP1 in various kinds of cancer cell lines.

### SPP1 mRNA Expressions in Normal Lung Tissues and LUAD Tissues

To compare the mRNA expression levels of SPP1 in LUAD with normal lung tissues, Gene Expression Omnibus (GEO^2^) database was employed for analyses. The mRNA expression levels were shown as the intensity of log_2_ median centered, and the SPP1 mRNA expression levels in LUAD tissues were compared with that in control normal lung tissues by Mann–Whitney *U* test. There were seven datasets that were used to compare the SPP1 expression in normal lung tissue and LUAD, including GSE2514 (20 LUAD samples and 19 normal lung tissue samples) (Stearman et al., 2005), GSE7670 (20 LUAD samples and 30 normal lung tissue samples) (Su et al., 2007; Chen et al., 2009), GSE10072 (58 LUAD samples and 49 normal lung tissue samples) (Landi et al., 2008), GSE19188 (45 LUAD samples and 65 normal lung tissue samples) (Hou et al., 2010), GSE31210 (226 LUAD samples and 20 normal lung tissue samples) (Okayama et al., 2012; Yamauchi et al., 2012), GSE32863 (58 LUAD samples and 58 normal lung tissue samples) (Selamat et al., 2012), and GSE83227 (132 LUAD samples and 17 normal lung tissue samples) (Bhattacharjee et al., 2001) from the GEO database. Each dataset was downloaded in Series Matrix File (s) format. The preprocess of each dataset adopted the original author’s preprocessing results.

### The Human Protein Atlas Analysis

The protein levels of SPP1 expression in LUAD samples and normal lung samples from the Human Protein Atlas (HPA)^3^ database were analyzed by the immunohistochemistry (IHC) staining data (Uhlen et al., 2005, 2010, 2015). These data were then used to analyze the correlation between the mRNA levels of SPP1 expression and prognosis. The expression levels could be divided into four categories: high, medium, low, and not detected by the scoring system, which consists of the proportion of stained cells (>75, 25–75, or <25%) and the intensity of staining (strong, moderate, weak, or negative).

### Data Mining From the GEO Database

GSE68465, including 19 normal lung tissues and 443 LUAD samples, was downloaded from the GEO database. GSE68465 was performed on GPL96 (Shedden et al., 2008). A large amount of clinicopathological information, such as gender, age, and tumor–node–metastasis (TNM) stage, was collected from the database as well. The associated probe for SPP1 was 209875_s_at. We then analyzed the survival information of samples with GSE68465 using the Kaplan–Meier method. GSE68465 were normalized by the MAS5 algorithm and log_2_ transformation were conducted.

### TIMER Analysis

Tumor Immune Estimation Resource (TIMER)^4^ systematically analyzed the comprehensive resource of immune infiltrates in multiple tumor types, including six types of immune infiltrates (CD4^+^ T cells, B cells, CD8^+^ T cells, macrophages, neutrophils, and dendritic cells) (Li et al., 2016; Li T. et al., 2017). We selected the parameters: “Gene,” gene symbol: “SPP1,” cancer types: “LUAD (lung adenocarcinoma),” immune infiltrates: “Default” to explore the relationship between the SPP1 expression and the proportion of immune infiltration. We selected the parameters “survival,” cancer types: “LUAD (Lung Adenocarcinoma),” clinical: (optional): “all,” Immune infiltrates: “Default,” gene symbol: “SPP1” to conduct survival analysis and build a multivariable Cox proportional hazards model. This model was fitted by the function coxph() using R package “survival.” Baseline variables considered to be univariate with outcome or clinically relevant to the results were included into multivariable Cox proportional hazards model (Stone et al., 2011).

### Gene Set Enrichment Analysis

Gene Set Enrichment Analysis (GSEA) was used for gene enrichment analysis (Mootha et al., 2003; Subramanian et al., 2005). GSE68465 was included in GSEA, and lung cancer tissue samples were divided into low- and high-expression groups according to the median expression level of SPP1. The hallmark gene set in MSigDB database was used as reference gene set. The default weighted enrichment statistical method was applied to enrichment analysis, and the number of the gene set permutation for each analysis was set as 1,000 times. Pathways with false discovery rate (FDR) *q* < 0.25 and nominal *P* < 0.05 were considered as significantly enriched pathways.

### Validation in the Cancer Genome Atlas Dataset

FPKM-UQ normalized mRNA expression of The Cancer Genome Atlas (TCGA)-LUAD and clinical data were downloaded from the GDC Data Portal^5^, and then, we screened the samples based on the complete survival information and gene expression data. The TCGA-LUAD gene expression data were processed by log_2_ transformation to compare the expression levels of SPP1 in normal lung tissue samples (*N* = 59) and LUAD samples (*N* = 504). According to the expression level of SPP1, the samples above the median expression level were set as high-expression group (*N* = 252), and the samples below the median expression level were set as low-expression group (*N* = 252) (Tang et al., 2018). Then, we explored the relationship between the SPP1 expression level and the prognosis of LUAD.

### Statistical Analysis

Mann–Whitney *U* test was used to compare the SPP1 expression in LUAD tissues and normal lung tissues from the GEO database. The relevance between clinicopathological characteristics and the SPP1 mRNA expression level was analyzed by χ^2^ test with the SPSS software (version 20). For the LUAD samples in GSE68465, according to the expression level of SPP1, the samples above the median expression level were set as high-expression group (*N* = 222), and the samples below the median expression level were set as low-expression group (*N* = 221) (Tian et al., 2018; Guo et al., 2020). Using the GraphPad Prism software (version 8) and Kaplan–Meier method, the relationship between the overall survival rate and the SPP1 expression level was analyzed. Univariate Cox regression model was conducted using SPSS software (version 20). Statistical significance was set as *P* < 0.05.

## Results

### SPP1 mRNA Expressions in Normal Lung Tissues and Tumor

**FIGURE 1:**
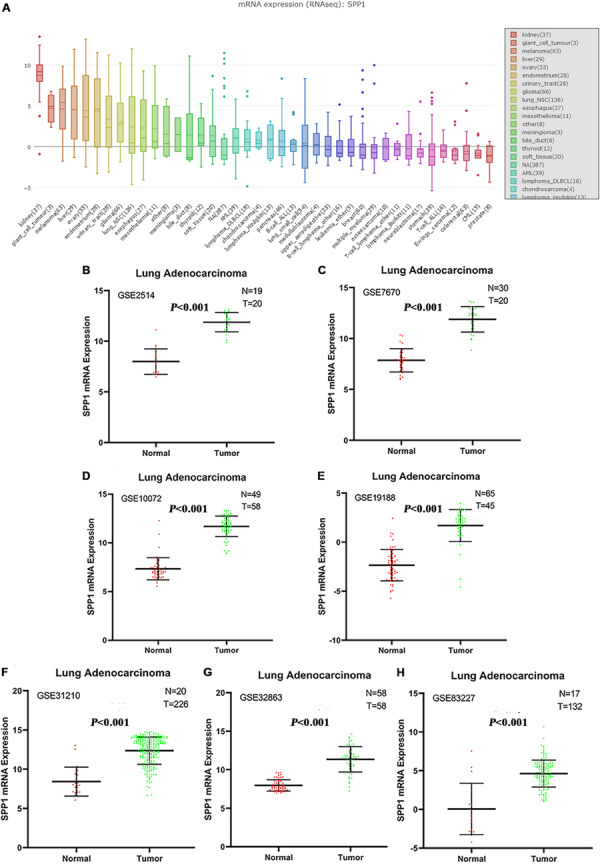
**(A)** The messenger RNA (mRNA) expression levels of SPP1 in non-small cell lung cancer (NSCLC) and other cancer cell lines from the Cancer Cell Line Encyclopedia (CCLE) database. NSCLC ranked ninth among cancer cell lines based on SPP1 expression. **(B–H)** Comparison of SPP1 mRNA levels in normal lung tissues and lung adenocarcinoma (LUAD) tissues across seven analyses of LUAD, and each study showed that the SPP1 mRNA expression levels in LUAD tissues were significantly higher than that in normal lung tissues.

NSCLC ranked ninth among cancer cell lines based on the SPP1 expression according to the data from CCLE database (Figure 1A). In seven studies, the SPP1 mRNA expression levels in LUAD tissues were significantly higher than that in normal lung tissues (*P* < 0.05). Each study supported the above conclusion (Figures 1B–H).

### SPP1 Expression at the Protein Level

**FIGURE 2:**
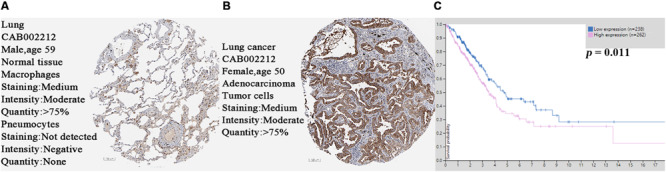
**(A)** The protein expression of secreted phosphoprotein 1 (SPP1) was detected in normal lung tissues from the Human Protein Atlas (HPA) database. **(B)** The protein expression of SPP1 was medium in lung adenocarcinoma (LUAD) from the HPA database. **(C)** The survival curves of 262 samples with high SPP1 mRNA expression and 238 samples with low SPP1 mRNA expression in HPA database.

Based on the protein expression data from the HPA, the protein expression of SPP1 in LUAD tissues and control normal lung tissues were compared by antibody CAB002212. The SPP1 protein expression in normal lung tissues was “not detected” in two cases. However, the protein expression of SPP1 in LUAD was “medium” in two cases, “low” in three cases, and “not detected” in one case (Figures 2A,B). It was further confirmed that SPP1 protein was highly expressed in LUAD than in normal lung tissues. The survival curves of 262 samples with high SPP1 expression and 238 samples with low SPP1 expression were obtained by analyzing the relevance between the SPP1 mRNA expression and prognosis in the HPA database (Figure 2C). It was found that compared with the SPP1 low-expression group, the overall survival rate of SPP1 high-expression group was significantly lower (log-rank *P* = 0.011).

### Association Between Clinicopathological Characteristics and SPP1 mRNA Expression

**FIGURE 3:**
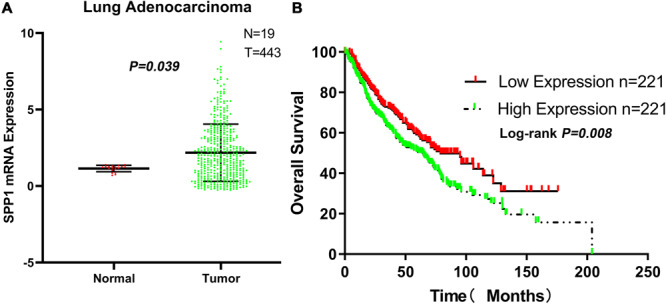
**(A)** The messenger RNA (mRNA) expression of secreted phosphoprotein 1 (SPP1) in lung adenocarcinoma (LUAD) and normal lung tissues based on GSE68465. **(B)** The overall survival rates of the SPP1 high-expression group and the SPP1 low-expression group in LUAD based on GSE68465.

The SPP1 mRNA expression level in LUAD tissues was significantly higher than that in normal lung tissues in GSE68465 (*P* = 0.039; Figure 3A). Based on the clinical and pathological information from GSE68465, the SPP1 expression in LUAD was not related to age, T stage, and smoking history (*P* > 0.05) but related to the gender, N stage, histological grade, and progression and relapse (*P* < 0.05; Table 1). In men, the SPP1 expression levels were higher compared to that in women. In the progression of lymph node metastasis of LUAD, the higher the N stage, the higher the SPP1 expression level. As LUAD progresses or relapses, the SPP1 expression could increase. In the pathological grade, the expression level of SPP1 was higher in lung adenocarcinoma samples whit moderately differentiated. Based on the clinical follow-up data of GSE68465, the overall 5-year survival rates of the SPP1 high-expression group and the SPP1 low-expression group were 50.574 and 59.181%, respectively [hazard ratio (HR) = 0.7057; 95% CI, 0.5467–0.9109; *P* = 0.008; Figure 3B]. By univariate Cox regression model, we concluded that age, gender, T stage, N stage, and SPP1 had an impact on overall survival rate of LUAD patients (*P* < 0.05; Supplementary Table S1).

**TABLE 1:** Associations between SPP1 expression and clinicopathological factors of patients with LUAD (based on GSE68465).

	**Total number**	**SPP1**			
**Parameters**	**of patients**	**expression**		**χ^2^**	***P* value**
		**Low**	**High**		
Gender	443			6.340	0.012
Male		98	125		
Female		123	97		
Age	443			0.435	0.510
<60 years		67	61		
≥60 years		154	161		
T stage	441			6.468	0.091
T1		72	78		
T2		133	118		
T3		14	14		
T4		2	10		
N stage	440			13.486	0.001
N0		158	141		
N1		48	40		
N2		14	39		
Progression or relapse	362			5.485	0.019
Yes		96	109		
No		93	64		
Histological grade	436			9.195	0.010
Well differentiated		40	20		
Moderate differentiation		93	116		
Poorly differentiated		84	83		
Smoking history	349			2.357	0.125
Yes		170	130		
No		22	27		

### The Relationship Between SPP1 and Immune Infiltrates

**FIGURE 4:**
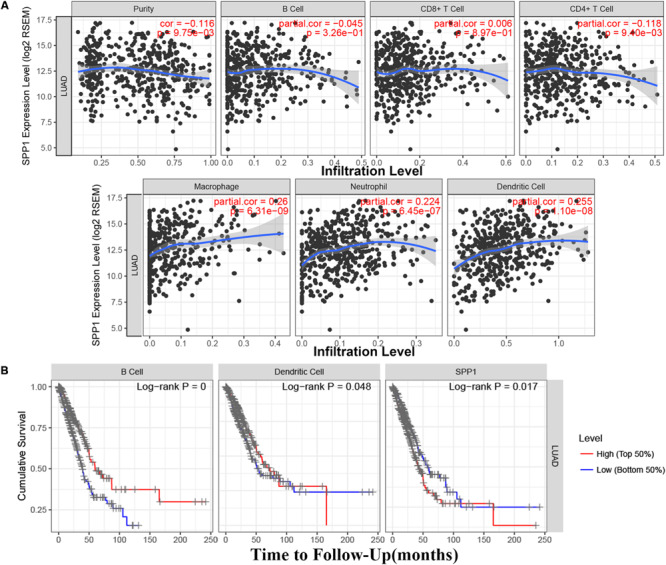
**(A)** The relationship between secreted phosphoprotein 1 (SPP1) expression and six immune infiltrates in lung adenocarcinoma (LUAD) from the Tumor Immune Estimation Resource (TIMER). **(B)** The survival rate of SPP1 and the LUAD immune subsets from the TIMER.

Based on the TIMER database, the relationship between SPP1 expression and CD4^+^ T cell, neutrophil, macrophage, and dendritic cell infiltration was weak in LUAD (*P* < 0.05; Figure 4A). According to the Kaplan–Meier plots for immune infiltration and SPP1 expression, the survival rate of high level of B cell and dendritic cell infiltration group was significantly higher than that of low level of infiltration group (*P* < 0.05). At the same time, the group with low SPP1 expression had significantly higher survival rates than that group with high SPP1 expression (*P* < 0.05; Figure 4B).

### SPP1 Is an Independent Prognostic Marker for LUAD Patients

The clinical relevance of LUAD immune subsets was explored by a multivariable Cox proportional hazards model, and SPP1 could be considered as an independent prognostic marker for LUAD patients (HR = 1.150; 95% CI, 1.048–1.261; *P* = 0.003; Table 2).

**TABLE 2:** Multivariate Cox proportional hazard regression analyses of the relationship between clinicopathological characteristics and overall survival in LUAD from the Tumor Immune Estimation Resource (TIMER).

**Variables**	**HR**	**95% CI**	***P* value**
Age	1.017	1.000–1.035	0.046
Gender (male)	0.826	0.591–1.154	0.262
Stage2	2.350	1.548–3.567	0.000
Stage3	2.999	1.969–4.569	0.000
Stage4	3.789	2.068–6.941	0.000
Purity	1.471	0.642–3.370	0.362
B_cell	0.040	0.003–0.612	0.021
CD8_Tcell	2.344	0.341–16.136	0.387
CD4_Tcell	36.137	2.255–579.010	0.011
Macrophage	0.588	0.035–9.888	0.712
Neutrophil	0.262	0.005–13.909	0.508
Dendritic	0.333	0.077–1.444	0.142
SPP1	1.150	1.048–1.261	0.003

### Gene Set Enrichment Analysis

**FIGURE 5:**
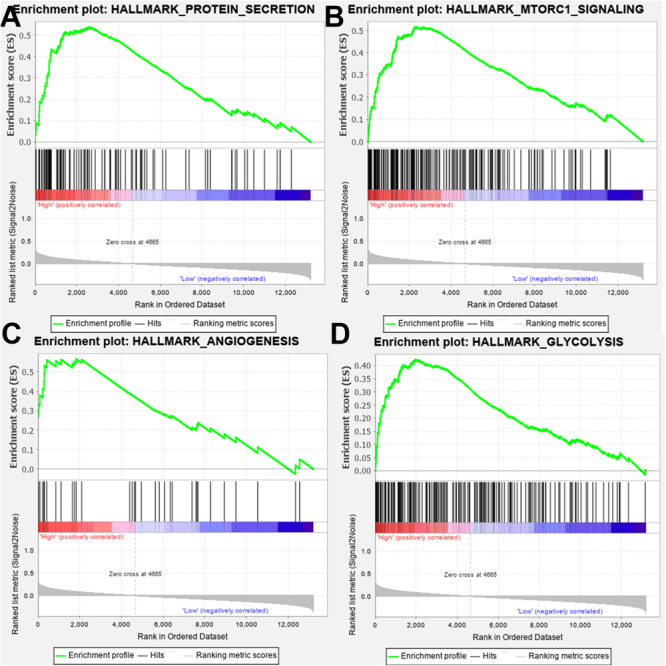
Gene set enrichment analysis (GSEA) showed that the samples with high secreted phosphoprotein 1 (SPP1) expression enriched in **(A)** protein secretion, **(B)** mTORC1 signaling, **(C)** angiogenesis, and **(D)** glycolysis pathway.

The effect of SPP1 expression on biological pathway was analyzed by GSEA based on GSE68465. The results of GSEA indicated that samples with high SPP1 expression enriched in protein secretion, mTORC1 signaling, angiogenesis, and glycolysis pathway (Table 3 and Figure 5).

**TABLE 3:** Gene set enrichment in SPP1 high-expression phenotype (based on GSE68465).

**Gene set name**	**Normalized enrichment score**	**False discovery Rate *q* value**	**Normalized *P* value**
PROTEIN_SECRETION	0.538	0.211	0.010
MTORC1_SIGNALING	0.518	0.127	0.006
ANGIOGENESIS	0.566	0.138	0.021
GLYCOLYSIS	0.422	0.202	0.020

### Validation in TCGA Dataset

**FIGURE 6:**
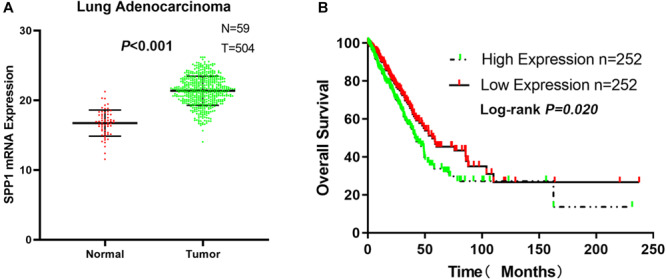
**(A)** The messenger RNA (mRNA) expression of secreted phosphoprotein 1 (SPP1) in lung adenocarcinoma (LUAD) and normal lung tissues based on The Cancer Genome Atlas (TCGA)-LUAD. **(B)** The overall survival rates of the SPP1 high-expression group and the SPP1 low-expression group in LUAD based on TCGA-LUAD.

From the TCGA-LUAD data, the SPP1 mRNA expression in normal lung tissue was significantly lower than that in LUAD samples (*P* < 0.001; Figure 6A). Based on the clinical follow-up data of GSE68465, the overall 5-year survival rates of the SPP1 high-expression group and the SPP1 low-expression group were 33.755 and 45.329%, respectively (HR = 0.7088; 95% CI, 0.5298–0.9483; log-rank *P* = 0.020; Figure 6B). According to the clinical and pathological information from TCGA-LUAD, the SPP1 expression in LUAD was related to the N stage. As the N stage progresses, the expression level of SPP1 is higher than before (*P* < 0.05; Supplementary Table S2). The analysis results obtained using TCGA-LUAD data were basically consistent with the results obtained using GSE68465.

## Discussion

SPP1 is a secreted calcium binding phosphorylation protein, which can activate many kinds of signaling pathways, promote tumor growth, metastasis, and angiogenesis and regulate the expression of various carcinogenic and angiogenic molecules. More and more studies have shown that SPP1 is closely related to the migration and metastasis of the malignant tumors. In multiple malignant tumors such as gastric, esophageal, and colorectal cancers, SPP1 could be detected to have a significantly high expression. Moreover, the SPP1 expression level was significantly higher in tumors with higher malignancy than that in tumors with lower malignancy (Lin et al., 2015; Xu et al., 2017; Chen et al., 2018; Assidi et al., 2019). Xu et al. (2017) found that SPP1 could activate the epithelial–mesenchymal transition (EMT) pathway to promote the metastasis of colorectal cancer. Chen et al. (2018) detected the potential functional marker single nucleotide polymorphism (tagSNP) of SPP1 by PCR and found that the frequency of SPP1 rs4754 genotype was significantly different from that of the control group, and the rs4754 TT genotype increased the risk of gastric cancer. Qin et al. (2018) found that SPP1 was upregulated in tumor tissues and plasma of patients with head and neck cancer, and the overexpression of SPP1 or the elevated level of plasma SPP1 in head and neck cancer tissues was associated with high malignancy and poor prognosis. These studies showed that SPP1 played a prominent role in the development of multiple tumors.

Giopanou et al. (2019) found that loss of SPP1 expression could be protective for mice harboring KRASG12D-driven LUAD; however, overexpression of SPP1 could promote the occurrence of early tumors and cause tumor-related inflammation. Zhang et al. (2017) detected that SPP1 was highly expressed in tumor-associated macrophages (TAMs) and LUAD tissues, and SPP1 played a role in lung cancer escape and mediating macrophage polarization. Wang et al. (2019) detected that inhibiting the SPP1 expression in lung cancer may overcome the resistance of second-generation EGFR TKI, and the expression of SPP1 may lead to increased resistance of lung cancer. These studies indicated that there was a close relationship with SPP1 and LUAD; SPP1 affecting the mechanism of lung adenocarcinoma needs to be further explored.

In this study, bioinformatics analysis revealed that SPP1 was highly expressed in LUAD tissues, which was related to poor prognosis. At the cell line level, NSCLC ranked ninth among cancer cell lines based on SPP1 expression. At the mRNA and protein level, we confirmed that SPP1 expression in LUAD tissues was higher than that in normal control based on the data from CCLE, GEO and HPA databases. We further verified the difference in SPP1 between LUAD and normal lung tissues, and the prognosis of LUAD samples by GSE68465. We found that the expression of SPP1 was related to gender, N stage, histological grade, and progression and relapse. Moreover, age, gender, T stage, N stage, and SPP1 had an impact on overall survival for LUAD patients. Then, we found that, compared to the SPP1 low-expression group, the overall survival rate of SPP1 high-expression group was significantly lower. The relationship between SPP1 expression and CD4^+^ T cell, macrophage, neutrophil, and dendritic cell infiltration was weak in LUAD from the TIMER database. Meanwhile, SPP1 could be considered as an independent prognostic marker for LUAD by a multivariable Cox proportional hazards model. The results indicated that samples with high SPP1 expression enriched in protein secretion, mTORC1 signaling, angiogenesis, and glycolysis pathway by GSEA based on GSE68465. The analysis results obtained using TCGA-LUAD data were basically consistent with the results obtained using GSE68465.

In the lung cancer microenvironment, tumor-infiltrating immune cells can not only attack and kill lung cancer cells to inhibit tumor progression but also screen tumor cells that were more suitable for survival in immune-active hosts or change the tumor microenvironment and promote tumor progression (Merlo et al., 2016). CD4^+^ T cells in the tumor microenvironment were divided into two subgroups of Th1 cells and Th2 cells according to the phenotype. The interaction between lung cancer cells, Th1 cells, and Th2 cells led to an imbalance of Th1/Th2 cell proportion, increasing the risk of lung cancer progression (Bremnes et al., 2016). Activated Th1 cells released cytokines such as tumor necrosis factor alpha (TNF-α), interferon gamma (IFN-γ), and interleukin (IL)-2, which can inhibit tumor progression by mediating cellular immunity, inducing apoptosis of tumor cells, and inhibiting the formation of neovascularization in tumors, while activated Th2 cells released IL-4, IL-5, IL-10, and IL-13 and other cytokines, through the mediator fluid immunity, and played a role in promoting tumor growth (Huang et al., 2017; Mateu-Jimenez et al., 2017). The M2 phenotype TAMs participated in complex autocrine and paracrine pathways and promoted tumor progression (Li et al., 2018). Dendritic cells, as powerful antigen-presenting cells, were the central link for initiating, regulating, and maintaining immune responses, and played a key role in inducing antilung cancer immune responses (Sabado et al., 2017). Zhang et al. (2017) detected that SPP1 was highly expressed in TAMs and LUAD tissues. Based on the above researches, the mechanism by which SPP1 promotes the development of LUAD may be closely related to changes in the immune microenvironment.

SPP1 may be involved in the secretion of exosomes of tumor cells. Tumor cells exchanged materials and information with the surrounding microenvironment by releasing a large number of exosomes, microbubbles or microparticles, and apoptotic bodies, which participated in promoting tumor growth and progression (Martins et al., 2013; McAllister and Weinberg, 2014). Excessive activation of mTOR signaling pathway was ubiquitous in tumor cells. mTOR activation played an important role in tumor cell growth, protein synthesis, cell metabolism, and other physiological and pathological processes, and its over activation could lead to the proliferation of a variety of cancer cells (Gweon and Kim, 2013; Martini et al., 2014; Safdari et al., 2015). Park et al. (2016) confirmed that resveratrol could induce autophagy of breast cancer cell MCF-7 by inhibiting the expression of mTOR. Because of the rapid proliferation of tumor cells, the microenvironment was always in a relatively hypoxic state, which was the basis of pathological angiogenesis. Neovascularization provided oxygen and nutrition for tumor growth and transported and removed its metabolic waste, thus supporting tumor survival (Hattori et al., 2011). Tumor angiogenesis was the key to tumor growth, invasion, and metastasis (Folkman, 1971; Jain, 2013). Warburg (1928) found that normal cells chose oxidative phosphorylation to provide ATP, while tumor cells preferred glycolysis; even when oxygen was sufficient, tumor cells still provided energy through glycolysis. The change in glycometabolism pathway was one of the differences between tumor cells and normal cells. Tumor cells could produce more nucleotides, fatty acids, proteins, and ATP through enhanced aerobic glycolysis, which provided a material basis for the rapid proliferation of tumor cells (Lunt and Vander, 2011). At the same time, it could reduce the production of reactive oxygen species, improve the antioxidant capacity of cells, and reduce the apoptosis of cells. In addition, aerobic glycolysis could produce a lot of lactic acid, creating an acidic microenvironment for tumor cells, which was conducive to the invasion and metastasis of tumor cells (Lu et al., 2015). Based on the above researches, SPP1 may be involved in protein secretion, mTORC1 signaling, angiogenesis, and glycolysis pathways, which in turn affect the occurrence and development of LUAD.

Although there is no research confirmed at present, according to the results of bioinformatics analysis, it can be concluded that SPP1 has the potential to be a new target for molecular targeted therapy of LUAD. However, due to the lack of necessary experimental conditions, the specific mechanism of SPP1 in the development of LUAD has not been thoroughly explored. The research group will continue to explore the specific role of SPP1 in the development of LUAD after the experimental conditions are perfect.

## Conclusion

To sum up, we found that SPP1 was highly expressed in LUAD, which was closely related to the clinical characteristics of LUAD patients. Compared with the SPP1 low-expression samples, the overall survival rate of the SPP1 high-expression samples was significantly lower. At the same time, we explored the relationship between SPP1 and immune infiltration and elucidated that SPP1 enriched in protein secretion, mTORC1 signaling, angiogenesis, and glycolysis pathway. Therefore, SPP1 can not only affect the occurrence and development of LUAD but also be an independent prognostic marker of LUAD. SPP1 is expected to become a new target for molecular targeted therapy.

## Data Availability Statement

All datasets generated/analyzed for this study are included/have their accession numbers included in the article/Supplementary Material.

## Author Contributions

WH and SL conceived and designed the study. ZG, JH, and YW performed the analysis procedures. JH, ZG, WL, and X-PL analyzed the results. JY, WH, and SL contributed to the analysis tools. ZG and JH contributed to the writing of the manuscript. All authors have reviewed the manuscript.

## Conflict of Interest

The authors declare that the research was conducted in the absence of any commercial or financial relationships that could be construed as a potential conflict of interest.

## Footnotes

1 https://portals.broadinstitute.org/ccle/

2 http://www.ncbi.nlm.nih.gov/geo/

3 http://www.proteinatlas.org/

4 https://cistrome.shinyapps.io/timer/

5 https://portal.gdc.cancer.gov/
