# Supplementary material for: Analysis of Expression and Its Clinical Significance of the Secreted Phosphoprotein 1 in Lung Adenocarcinoma
Source: Front Genet. 2020 Jun 12;11:547. doi: 10.3389/fgene.2020.00547 (PMC7303289; doi:10.3389/fgene.2020.00547)
Supplement: Supplementary file 1 [file Table_1.DOCX]

**Table S1: Univariate Cox Proportional Hazard Regression Analyses of the Relationship Between Clinicopathologic Characteristics and Overall Survival in LUAD (based on GSE68465)**

| Variables | Hazard Ratio | *P* Value |
| --- | --- | --- |
|  | (95% Confidence Interval) |  |
| Age |  |  |
| <60 years | Reference | 0.002 |
| ≥60 years | 1.627(1.200-2.205) |  |
| SPP1 |  |  |
| Low | Reference | 0.008 |
| High | 1.42(1.096-1.839) |  |
| Gender |  |  |
| Male | Reference | 0.013 |
| Female | 0.719(0.555-0.932) |  |
| T Stage |  |  |
| T_1_ | Reference | <0.001 |
| T_2_ | 1.463(1.088-1.967) | 0.012 |
| T_3_ | 3.310(2.054-5.336) | <0.001 |
| T_4_ | 4.257(2.174-8.338) | <0.001 |
| N Stage |  |  |
| N_0_ | Reference | <0.001 |
| N_1_ | 2.273(1.670-3.093) | <0.001 |
| N_2_ | 3.892(2.767-5.474) | <0.001 |
| Progression or Relapse |  |  |
| NO | Reference | <0.001 |
| YES | 5.196(3.594-7.513) |  |
| Histologic Grade |  |  |
| Well Differentiated | Reference | 0.428 |
| Moderate Differentiation | 1.109（0.728-1.691） | 0.630 |
| Poorly Differentiated | 1.278（0.832-1.962） | 0.263 |
| Smoking History |  |  |
| NO | Reference | 0.344 |
| Yes | 1.246(0.790-1.965) |  |
