# Supplementary material for: Analysis of Expression and Its Clinical Significance of the Secreted Phosphoprotein 1 in Lung Adenocarcinoma
Source: Front Genet. 2020 Jun 12;11:547. doi: 10.3389/fgene.2020.00547 (PMC7303289; doi:10.3389/fgene.2020.00547)
Supplement: Supplementary file 2 [file Table_2.DOCX]

**Table S2: Associations Between SPP1 Expression and Clinicopathological Factors of Patients with LUAD (based on TCGA-LUAD)**

| Parameters | Total Number of Patients | SPP1 Expression | | χ2 | *P* value |
| --- | --- | --- | --- | --- | --- |
|  |  | Low | High |  |  |
| Gender | 504 |  |  | 0.032 | 0.858 |
| Male |  | 116 | 118 |  |  |
| Female |  | 136 | 134 |  |  |
| Age | 494 |  |  | 2.780 | 0.096 |
| ＜55 years |  | 42 | 29 |  |  |
| ≥55 years |  | 205 | 218 |  |  |
| T Stage | 501 |  |  | 0.969 | 0.809 |
| T1 |  | 89 | 79 |  |  |
| T2 |  | 130 | 139 |  |  |
| T3 |  | 22 | 23 |  |  |
| T4 |  | 9 | 10 |  |  |
| N Stage | 492 |  |  | 13.593 | 0.004 |
| N0 |  | 180 | 145 |  |  |
| N1 |  | 35 | 59 |  |  |
| N2 |  | 30 | 41 |  |  |
| N3 |  | 0 | 2 |  |  |
| M Stage | 360 |  |  | 0.104 | 0.747 |
| M0 |  | 163 | 172 |  |  |
| M1 |  | 13 | 12 |  |  |
